# Supplementary material for: Isolation of Lactococcus lactis from Whole Crop Rice and Determining Its Probiotic and Antimicrobial Properties towards Gastrointestinal Associated Bacteria
Source: Microorganisms. 2021 Dec 3;9(12):2513. doi: 10.3390/microorganisms9122513 (PMC8703902; doi:10.3390/microorganisms9122513)
Supplement: Supplementary file 1 [file microorganisms-09-02513-s001.zip › microorganisms-1494093-supplementary (1).pdf]

microorganisms

Supplementary materials

Table S1. Physiochemical features of identified isolates RWP-3 and RWP-7.

Code	Stain	motile	shape	Catalase	Growth Condition	Strain	species
RWP-3	Positive	Non	Cocci	Negative	Facultative anaerobic	*Lactococcus*	*lactis*
RWP-7	Positive	Non	Cocci	Negative	Facultative anaerobic	*Lactococcus*	*lactis*

Table S2. Different carbohydrate substrates fermentation by RWP-3 and RWP-7.

S.No	Substrates	RWP-3	RWP-7
1	Glycerol	++	++
2	Erythritol	+	NF
3	D-Arabinose	+	NF
4	L-Arabinose	++	++
5	D-Ribose	+++	++
6	D-Xylose	+++	+++
7	L-Xylose	+++	+++
8	D-Adonitol	+	NF
9	Methyl- ß-D-xiloside	+	NF
10	D-Galactose	++	++
11	D-Glucose	+++	+++
12	D-Fructose	+++	+++
13	D-Mannose	+++	+++
14	L-Sorbose	+	NF
15	L-Rhamnose	++	+
16	Dulcitol	+	+
17	Inositol	+	NF
18	D-Mannitol	++	NF
19	D-Sorbitol	NF	NF
20	Methyl- αD-mannoside	NF	+
21	Methyl- α-D-glucoside	NF	+
22	N-acetyl glucosamine	+++	+
23	Amygdalin	++	NF
24	Arbutin	+++	+++
25	Esculin ferric citrate	+++	++
26	Salicin	+++	+++
27	D-Celiobiose	+++	+++
28	D-Maltose	+++	+++
29	D-Lactose	++	NF
30	D-Melibiose	++	+
31	D-Saccharose	+++	+++
32	D-Trehalose	++	NF
33	Inulin	++	++
34	D-Melezitose	NF	NF
35	D-Raffinose	++	NF
36	Amidon	+	++
37	Glycogen	++	NF
38	Xylitol	++	NF
39	Gentiobiose	++	NF
40	D-Turanose	NF	NF

41	D-Lyxose	NF	NF
42	D-Tagatose	++	NF
43	D-Fucose	+	NF
44	L-Fucose	+	NF
45	D-Arabitol	+	NF
46	L-Arabitol	+	NF
47	Potassium gluconate	++	NF
48	Potassium 2-Ketogluconate	+++	++
49	Potassium 5-Ketogluconate	NF	++

+ Low; ++ medium; +++ strong fermentation; NF: Not fermented.

Table S3. Production of extra cellular enzyme by RWP-3 and RWP-7.

**S.No**	**Enzymes**	**RWP3**	**RWP7**
1	Control	0	0
2	Alkaline phosphatase	+++	+++
3	Esterase (C_4)	+++	+++
4	Esterase lipase (C_8)	+++	+++
5	Lipase (C_{14})	++	++
6	Leucine arylamidase	+++	+++
7	Valine arylamidase	++	++
8	Cystine arylamidase	++	++
9	Trypsin-like serine protease	++	0
10	α-Chymotrypsin	++	0
11	Acid phosphatase	+++	+++
12	Naphthol-AS-biphosphohydrolase	+++	+++
13	α-Galactosidase	++	++
14	β-Galactosidase	++	++
15	β-Glucuronidase	0	0
16	α-Glucosidase	0	0
17	β-Glucosidase	0	0
18	n acetyl β glucosminidase	0	0
19	α-Mannosidase	0	0
20	α-Fucosidase	0	0

++ Medium; +++ strong production.

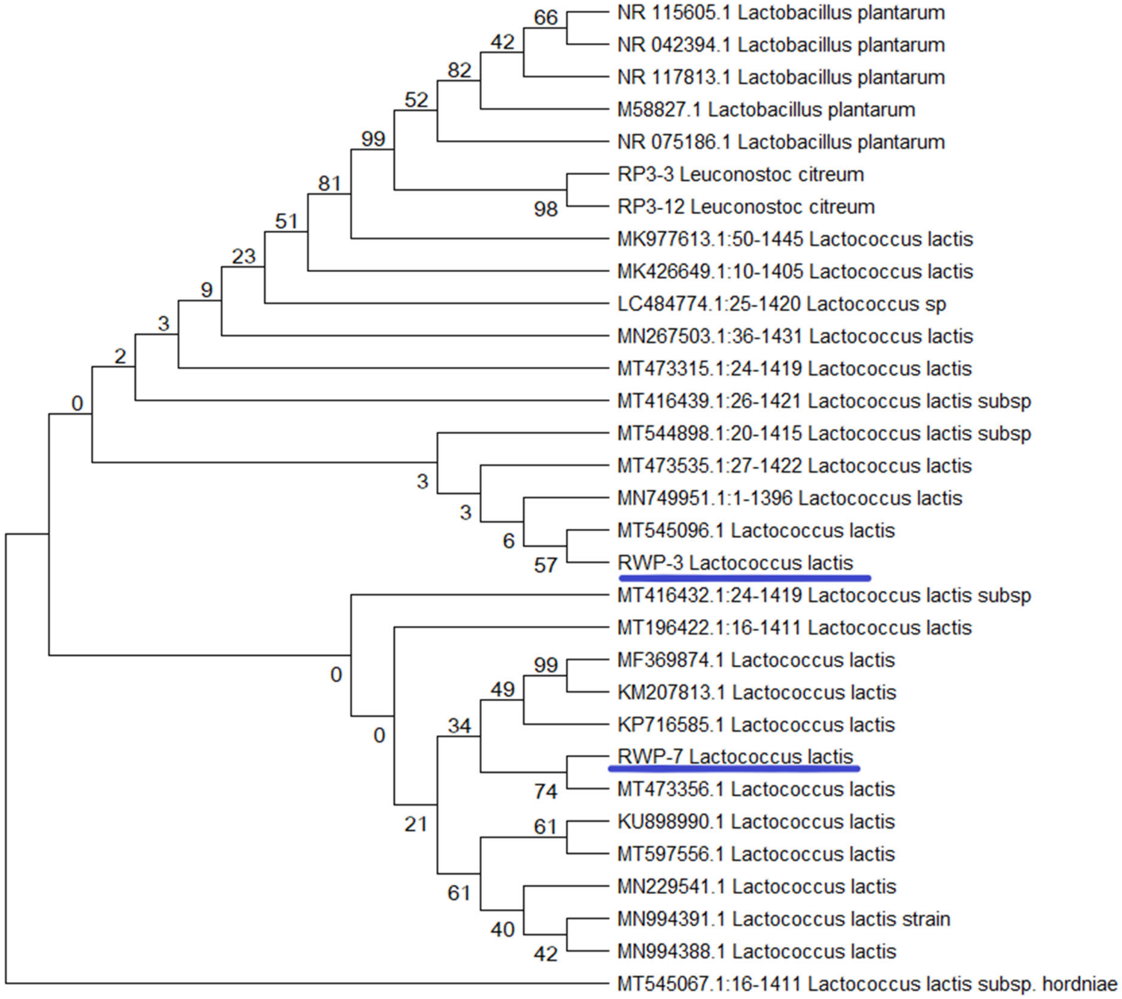

Figure S1. Phylogenetic tree based on 16srRAN sequences, showing the relationship between different *Lactococcus lactis* and other *Lactobacillus sp,* constructed by the neighbor-joining method using Molecular Evolutionary Genetics Analysis -11 (Mega-11 version).
